# Effects of Nitrite Stress on Growth, Glycolipid Metabolism, and Hepatic Metabolome in Spotted Seabass (*Lateolabrax maculatus*) Under High-Temperature Conditions

**DOI:** 10.3390/ani15131870

**Published:** 2025-06-24

**Authors:** Juan Gao, Shi Cao, Chen Shen, Jian Zhang, Ling Wang, Xueshan Li, Kangle Lu, Chunxiao Zhang, Kai Song

**Affiliations:** Laboratory of Aquatic Animal Nutrition, Fisheries College, Jimei University, Xiamen 361021, China; gaojuan3233@126.com (J.G.); 18554908325@163.com (S.C.); xiaobao991120@163.com (C.S.); zj19971024@126.com (J.Z.); lingwang@jmu.edu.cn (L.W.); xsli@jmu.edu.cn (X.L.); lukangle@jmu.edu.cn (K.L.); cxzhang@jmu.edu.cn (C.Z.)

**Keywords:** spotted seabass, nitrite stress, high temperature, growth, glycolipid metabolism

## Abstract

Nitrite is a common pollutant in aquaculture systems and can pose serious threats to fish health, especially under high-temperature conditions. This study aimed to investigate the impact of nitrite stress on the growth, glycolipid metabolism, and hepatic metabolomic profiles in the spotted seabass fry (*Lateolabrax maculatus*) under elevated temperature conditions at 33 °C. A total of 450 fish (28.52 ± 0.84 g) were randomly distributed into nine tanks and exposed to three nitrite concentrations (0, 8, and 16 mg/L), with samples collected on days 1, 3, 7, 14, 21, and 28. Results showed that higher nitrite levels significantly reduced final body weight, weight gain, survival rate, hepatosomatic index, and viscerosomatic index. Blood glucose and triglyceride levels, whole-body crude lipid, liver total cholesterol, and hepatic glycogen content also declined significantly under higher nitrite stress. In contrast, hepatic lactate and lactate dehydrogenase increased in the high-nitrite group. Gene expression analysis revealed suppressed lipid synthesis and enhanced lipolysis under nitrite exposure. Metabolomic analysis further demonstrated disruptions in key energy-related pathways, including the TCA cycle, pentose phosphate pathway, and insulin signaling. These findings indicate that nitrite stress impairs growth and energy metabolism in spotted seabass, which respond by mobilizing energy reserves to cope with combined stress of high temperature and nitrite.

## Simple Summary

This study investigated how nitrite, a common pollutant in aquaculture water, affects the health and growth of the spotted seabass (*Lateolabrax maculatus*) when they are raised in high-temperature conditions at 33 °C (the optimal keeping temperature range of 26–27 °C). The fish were exposed to different levels of nitrite, and their growth, survival, and changes in their liver and blood were tracked over four weeks. The results showed that higher nitrite levels caused the fish to grow more slowly, have lower survival rates, and store less lipid and sugar in their bodies. Their livers showed signs of stress, with more substances related to energy breakdown and fewer signs of fat production. Key genes responsible for storing and using energy were also affected. Detailed analysis of the liver showed that important energy pathways were disrupted. Overall, the study found that nitrite stress makes it harder for the spotted seabass to grow and survive in warm water by forcing their bodies to break down fats and produce sugar to meet energy needs. These findings help fish farmers understand the risks of poor water quality and can guide better practices to protect fish health, especially as temperatures rise due to climate change.

## 1. Introduction

Nitrite is a pervasive contamination in aquaculture systems. It mainly arises from the breaking down of organisms like residual bait and feces, as well as the incomplete nitrification, and it is one of the intermediate products in the process of nitrogen cycling [1,2]. Intensive aquaculture systems exhibit dangerously elevated nitrite concentrations (10–99 mg/L NO_2_^−^) that far exceed natural water levels (<1 mg/L), creating urgent threats to fish health and production viability [3]. When excessive cumulative nitrite in the water column exceeds the tolerance range of aquatic animals, it will disrupt physiological processes, including oxygen transport, ion regulation, and metabolic balance [4,5]. Furthermore, the intensive aquaculture practices often expose fish to multiple stressors, including elevated water temperatures and increased concentrations of harmful substances such as nitrite [6,7,8,9,10]. Climate change-induced temperature rises are projected to exacerbate nitrite toxicity in aquaculture systems by increasing its accumulation. Multiple stressors pose cross-threats to fish health and are a serious impediment to the sustainable development of aquaculture [6].

The spotted seabass *Lateolabrax maculatus* is a commercially important marine fish [11]. Widely cultured along the coasts of China and Southeast Asia, this species exhibits high market value and robust environmental adaptability, with an optimal keeping temperature range of 26–27 °C [12,13]. The seasonal warmth in Southern China pushes water temperatures above 30 °C during summer, resulting in reduced growth performance, longer culture cycles, and increased costs, and even triggering regional industrial losses for the spotted seabass. In addition, temperature is also a critical determinant affecting the uptake and discharge of nitrite in fish. Elevated temperature accelerates the metabolic rate of the spotted seabass, increases oxygen demand, and exacerbates its toxicity by promoting nitrite uptake and disrupting osmoregulation [14,15,16]. Therefore, in practice, the cross-impacts of high temperature and nitrite stress seriously threaten the growth and survival of the spotted seabass, resulting in economic losses and resource wastage in the long run [17,18].

The hepatic system represents a pivotal detoxification organ for fish, where nitrites can be converted to the much less toxic nitrates, which are excreted in the form of urine and bile [19]. Moreover, the hepatic system acts as the main regulator of maintaining metabolic homeostasis, including the regulation of glucose and lipid metabolism [20,21]. Under stress conditions, the liver typically undergoes significant metabolic reprogramming, shifting energy expenditure from growth to survival to meet increased energy demands and reduce the deleterious ramifications of environmental pressures [22,23,24]. However, prolonged exposure to nitrite and high temperatures results in metabolic burden in fish metabolic organs such as the liver, where energy-allocation patterns are disrupted, causing metabolic imbalances that ultimately lead to reduced growth performance, abnormal glucose and lipid metabolism, impaired immune function, and increased mortality [16,25,26]. Nitrite and high temperature induce hypoxia, reprogram glucose and lipid metabolism, and disrupt the oxidative/osmotic balance, forcing energy from growth to stress compensation, resulting in changed weight gain and blood glucose/lipids [9,12,14,16]. However, no studies have yet been conducted using metabolomics to systematically analyze the synergistic toxicity mechanism of high temperature and nitrite stress on spotted sea bass. Therefore, it is necessary to explore the adaptive regulation of the spotted seabass liver to nitrite exposure and high temperature conditions, and to deeply analyze the importance of hepatic metabolic pattern transformation in fish stress physiology. This pioneering study employed physiological indicators and integrated metabolomics to uncover the synergistic impacts of high temperature and nitrite stress on the spotted seabass, overcoming limitations of conventional single-stressor approaches, identifying critical metabolic pathway disruptions and establishing predictive biomarkers for sustainable aquaculture practices.

This study examined how nitrite exposure at 33 °C influences growth performance, blood biochemistry, and liver glycolipid metabolism of the spotted seabass under a hyperthermic condition of 33 °C. The purpose was to gain an overarching overview of the growth and metabolism physiological responses of the spotted seabass to nitrite and heat stress. The results could inform subsequent development of nitrite stress-resistant diets to improve the sustainability and productivity of the spotted seabass farming.

## 2. Materials and Methods

### 2.1. Nitrite Stress Experiment

Based on the pre-tests, the 96 h Lethal Concentration 50% (LC50) for nitrite in the spotted seabass was obtained to be 80 mg/L [15]. Nitrite stress concentrations in this experiment were determined based on a 10% 96 h LC50 value. It is aimed at studying chronic/sub-lethal effects rather than acute death. The concentration gradients were therefore 0 mg/L (control group, measured value: 1 ± 0.5 mg/L), 8 mg/L (medium concentration group, measured value: 8 ± 1 mg/L), and 16 mg/L (high concentration group, measured value: 16 ± 1 mg/L). Sodium nitrite stock solution was prepared by dissolving high-purity NaNO_2_ (≥99.0%, Sigma-Aldrich, Shanghai, China) in deionized water. This stock solution was stored in a dark glass bottle at 4 °C to prevent photodegradation and used for daily nitrite concentration adjustments in the experimental tanks. This solution was replaced weekly to ensure stability. Sodium nitrite stock solution (10 g/L) was used to maintain constant nitrite levels, with daily monitoring and readjustment of culture water concentrations. After daily cleaning, exchange 20% of the water with pre-tempered fresh water at 33 ± 1 °C (chronic sub-lethal temperature stress for the spotted seabass). After cleaning, take a water sample collected from mid-water column for nitrite concentration measurement, and then adjust the relevant concentration calculation. Then, drop the reserve solution near the inlet and mix it with aeration for 15–30 min to ensure homogeneity for final verification. Measure the nitrite concentration again to reach 0 mg/L, 8 ± 1 mg/Land, and 16 ± 1 mg/L. Nitrite concentrations were determined using the UV-1200 ultraviolet–visible spectrophotometer according to the national standard method for nitrite determination [16]. Standardized water quality parameters were maintained throughout the experiment: temperature of 33 ± 1 °C, monitored with heating rod (Ningbo Sunsun Co., Ningbo, China) and a calibrated digital thermometer (Omega Engineering, Stamford, Connecticut, USA); dissolved oxygen of 5 ± 1 mg/L, measured using an optical DO sensor of the YSI EXO2 multi-parameter water quality analyzer; NH3-N < 0.1 mg/L, analyzed by the UV-1200 spectrophotometer via the Salicylic Acid Method (HJ 536-2009); and pH of 7.5–8.5, measured with the pH electrode of the YSI EXO2 analyzer.

### 2.2. Feeding Management

The experimental spotted seabass were purchased from a hatchery in Zhangzhou City, Fujian Province, China. The fish were then transported to the aquatic experimental field of Jimei University and acclimated in 1000 L culture tanks with dechlorinated tap water (freshwater). The fish were acclimated with commercial feed for 14 days, and when the fish were adapted to the study parameters, 450 fish (initial body weight 28.52 ± 0.84 g) with robust body and homogeneous morphology. Fish were randomly distributed among nine 200 L tanks (50 fish/tank, *n* = 3 replicates per treatment). Following acclimation, the water temperature was gradually increased (1 °C/day) to 33 °C. The fish were acclimated at 33 °C for 7 days before initiating nitrite exposure. Twice-daily feeding at 8:00 and 18:00 took place with a diet (47.26% protein, 12.34% lipid) ad libitum feeding (feeding to satiation) [16]. After 30 min feeding periods, the tanks were cleaned by siphoning residual feed and waste, with subsequent feed intake quantification. Mortalities were tracked daily, including individual weight measurements.

### 2.3. Sample Collection

Six temporal sampling points (days 1, 3, 7, 14, 21, and 28) were established, with seven fish randomly sampled per interval to record body length and weight, which were used to calculate the weight-gain rate, survival rate, and condition factor. Then, blood was collected from the tail, and five of them were placed in 2 mL enzyme-free centrifuge tubes and then centrifuged (at 3500 r/min, 4 °C, 10 min) to separate the serum after 24 h of resting in a 4 °C refrigerator. Then, the serum placed in a −80 °C refrigerator for storage in portions, which were used to measure the biochemical indexes of the serum. Seven fish were weighed in terms of viscera mass, liver, and abdominal fat for calculation of hepatosomatic index (HSI), viscerosomatic index (VSI), and abdominal-fat percentage (AFR). Liver samples from five fish were immediately frozen in 5 mL cryotubes and kept in a −80 °C refrigerator for measurement of hepatochemical indices and metabolomics analysis. After the 28th day of stress, triplicate whole-fish samples per tank were cryopreserved at −20 °C for composition analysis.

### 2.4. Chemical Composition

Proximate composition (moisture by 105 °C drying, crude protein via Dumas combustion, and crude lipid via Soxhlet extraction) was determined following the [27] methods.

### 2.5. Biochemistry Analyses

Biochemical parameters (T-CHO: total cholesterol assay kit, A111-1-1, COD-PAP method, microplate method; TG: triglyceride assay kit, A110-2-1, GPO-PAP enzyme method, single reagent spectrophotometric method; Glu: Glucose Assay Kit, F006-1-1; glucose oxidase method, microplate method) in serum were measured with standardized commercial kits (Nanjing Jiancheng Biotechnology Co., Ltd., Nanjing, China) in accordance with the provided protocols. This refers to the method in [20].

Liver tissue mass (g): Phosphate buffer = 1:9 was added to phosphate buffer and ground into tissue homogenate using an ultrasonic cell disruptor (ultrasonic homogenizer) at 60 Hz for 30 s. Then, the supernatant (2500 rpm, 10 min) was centrifuged in a high-speed centrifuge for measurement. Related methods refer to previous research [7,10,22,24]. Determination of T-CHO content, TG content, hepatic glycogen (GLY), hepatic lactate dehydrogenase (LDH), and hepatic lactic acid (LD) in liver was operated with the instruction manual of Nanjing Jiancheng Bioengineering Institute.

### 2.6. Expression of Genes Involved in Hepatic Glycolipid Metabolism

RNA extraction and RNA reverse transcription of cDNA and qPCR were performed using the kits provided by Nanjing Novozymes Biotechnology Co. (Nanjing, China). RNA integrity and concentration were analyzed by electrophoresis and spectrophotometry (ND-1000, Thermo Fisher Scientific, Wilmington, DE, USA). Outcomes are represented as relative expressions, the primers were prepared using Premier 5.0 software for sequence design, and the primer synthesis was performed by Shanghai Bioengineering Co. (Shanghai, China). Table 1 lists all primer sequences used in the experiments. The housekeeping gene β-actin was employed for normalization [20,23]. Acc1, Hmgcr, Chrebp, Atgl, Hsl, and Ppara were the target genes. The 2^−ΔΔCT^ calculation was applied for relative quantification. Thermal-cycling parameters: stage 1, 95 °C for 30 s (initial denaturation); stage 2, 40 cycles of 95 °C for 5 s (denaturation), 60 °C for 30 s (annealing/extension); and stage 3, melt curve analysis (65–95 °C, 0.5 °C increments). Amplification was performed on a QuantStudio 6 Pro Real-Time PCR System (Applied Biosystems, Carlsbad, CA, USA). Five biological replicates per timepoint were analyzed, with each sample run in technical triplicate [28].

### 2.7. Metabolomics

Determining the metabolomics of liver samples was entrusted to Guangzhou Kidio Biotechnology Co. (Guangzhou, China). The main steps included sample preparation, quality control standard generation, LC-MS/MS analysis, and subsequent data processing. The database was provided by Guangzhou Kidi Biotechnology Co., Ltd. (Guangzhou, China). Quality control standards specifically included dual-ionization mode QC, mixed samples, multilevel validation, system suitability test, process blanks for background subtraction, and PCA-based QC. Sample portions measuring 100 mg each were combined with 1 mL of refrigerated methanol/acetonitrile/water (2:2:1, *v*/*v*) solution for metabolite extraction, homogenized, and broken up with an MP homogenizer (24 × 2, 6.0 M/S, 20 s, 3 times). Samples were subjected to ultrasonication at low temperature for 30 min (repeated twice), followed by incubation at −20 °C for 60 min and centrifugation at 13,000× *g* for 15 min at 4 °C, and then the supernatant was taken (dispensed as 900 μL/tube). Following vacuum drying, the lyophilized samples were preserved at −80 °C for long-term storage. Prior to LC-MS analysis, the samples were reconstituted in 100 μL of 50% aqueous acetonitrile solution (*v*/*v*). After vigorous vortexing for complete dissolution, the samples underwent centrifugation at 14,000× *g* (15 min, 4 °C) to precipitate insoluble components. The resulting supernatant was then aliquoted into analytical vials for instrumental analysis. Separations were performed on an Agilent 1290 Infinity LC ultra-high-performance liquid chromatography (UHPLC) system equipped with an HILIC column. High-resolution mass spectrometry data were collected on an AB Sciex TripleTOF 6600 instrument configured for full-scan MS and data-dependent MS/MS acquisition. The acquired raw data files were converted to open-source. mzML format using ProteoWizard (v3.0.6428), followed by comprehensive metabolomic data processing with XCMS Online (v3.7.1) for feature detection, retention-time correction, and peak-area integration.

### 2.8. Statistical Analysis

All diets were administered in a strictly controlled randomized design. The Shapiro–Wilk and Kolmogorov–Smirnov variance tests demonstrated that all values were consistent with heteroscedasticity and normalism. Data were analyzed by one-way ANOVA (one-way ANOVA), using SPSS 21.0 statistical software, and multiple comparisons were performed using Duncan’s test, with a significant level of difference of *p* < 0.05. The experimental data were expressed as mean ± standard error (mean ± S.E.). Data labeled with different letters indicate statistically significant differences, whereas the same letters denote non-significant variations (*p* > 0.05).

## 3. Results

### 3.1. Growth Performance

**Figure 1:**
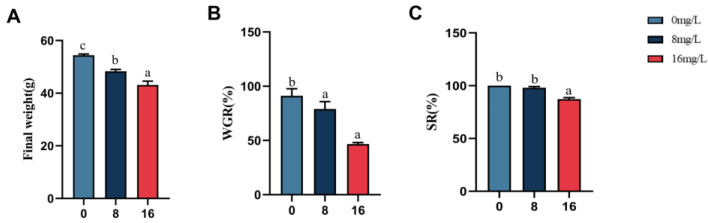
Growth performance of the spotted seabass under 33 °C nitrite stress. (**A**) Final weight, (**B**) weight-gain rate, and (**C**) survival rate. Bars with different letters indicate significant difference (*p* < 0.05). Value is mean ± S.E. (*n* = 3 for treatment). Weight-gain rate (WGR, %) = (final body weight–initial body weight)/initial body weight × 100. Survival rate (SR, %) = 100 × final fish number/initial fish number.

The effects of nitrite stress on the growth performance of the spotted seabass are presented in Figure 1. Following a 28-day nitrite treatment period, the final body weight of the spotted seabass exhibited a significant concentration-dependent decrease (*p* < 0.05). The 8 and 16 mg/L nitrite treatments significantly reduced WGR relative to controls (*p* < 0.05), with the 16 mg/L group also showing significantly lower survival relative to the 8 mg/L and control groups (*p* < 0.05). WGR in the 16 mg/L group decreased by 51% as opposed to controls.

### 3.2. Body Composition and Morphological Parameters

**Figure 2:**
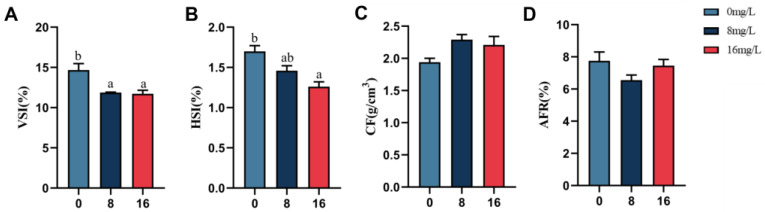
Body index of the spotted seabass under 33 °C nitrite stress. (**A**) Viscerosomatic index, (**B**) hepatosomatic index, (**C**) condition factor, and (**D**) abdominal-fat percentage. Bars with different letters indicate significant difference (*p* < 0.05). Value is mean ± S.E. (*n* = 3 for treatment). Viscerosomatic index (VSI, %) =100×visceral mass weight/final body weight. Hepatosomatic index (HSI, %) = 100 × liver weight/final body weight. Condition factor (CF, g/cm^3^) = 100 × final body weight/final body length. Abdominal-fat rate (AFR, %) = 100 × abdominal-fat weight/final body weight.

The impact of nitrite stress on the morphometric indices of the spotted seabass is demonstrated in Figure 2. The experimental results showed that the HSI and VSI of the spotted seabass decreased significantly with incremental nitrite supplementation after 28 days of nitrite stress (*p* < 0.05). The 16 mg/L nitrite-exposure group exhibited significantly reduced HSI and VSI values compared to controls (*p* < 0.05). Neither CF nor AFR showed significant intergroup variation (*p* > 0.05).

**Figure 3:**
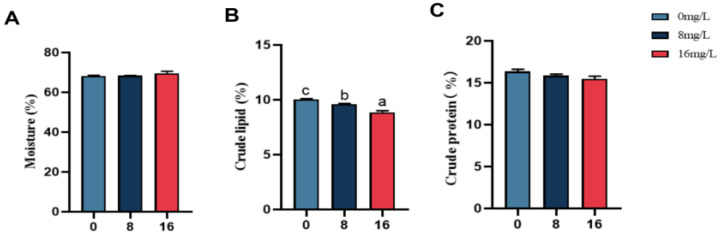
Whole-body composition of the spotted seabass under 33 °C nitrite stress. (**A**) Moisture (%), (**B**) crude lipid (%), and (**C**) crude protein (%). Bars with different letters indicate significant difference (*p* < 0.05). Value is mean ± S.E. (*n* = 3 for treatment).

Figure 3 illustrates the impact of nitrite exposure on body composition in the spotted seabass. Results indicated the crude lipid of the whole fish of the spotted seabass decreased significantly (*p* < 0.05) with elevated nitrite exposure, whereas different concentrations of nitrite stress revealed no treatment effects (*p* > 0.05) on the crude protein and moisture of the spotted seabass. The crude lipid content in the 16 mg/L group decreased by 12.03% as opposed to controls.

### 3.3. Serum Biochemical Indicators

**Figure 4:**
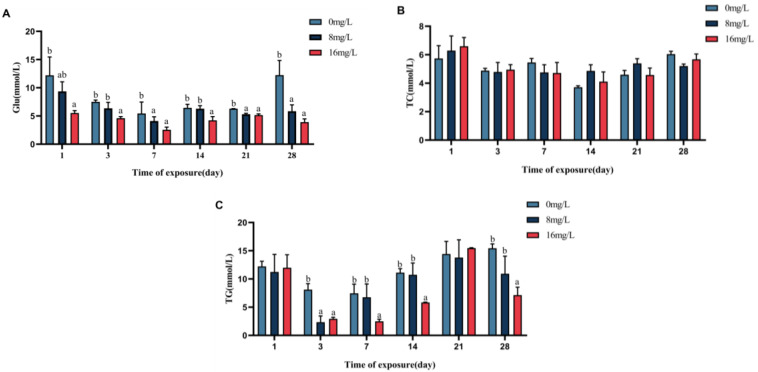
Blood biochemistry of the spotted seabass under 33 °C nitrite stress. (**A**) Glucose (Glu), (**B**) total cholesterol (TC), and (**C**) triglyceride (TG). Bars with different letters indicate significant difference (*p* < 0.05). Value is mean ± S.E. (*n* = 3 for treatment).

Changes in hematological Glu, TG, and TC contents of the spotted seabass are shown in Figure 4. The Glu content of the blood of the spotted seabass displayed progressive diminishment with elevated nitrite on days 1, 3, 7, 14, 21, and 28 (*p* < 0.05). In the 16 mg/L group, serum TG levels were significantly reduced on days 3, 7, 14, and 28 (*p* < 0.05), with no intergroup differences on days 1 and 21 (*p* > 0.05). The TC content of each nitrite concentration group was not significant (*p* > 0.05) on days 1, 3, 7, 14, 21, and 28 of stress. The hematological Glu in the 16 mg/L group decreased by 66.67%, as opposed to controls on days 28.

### 3.4. Liver Biochemical Indicators

**Figure 5:**
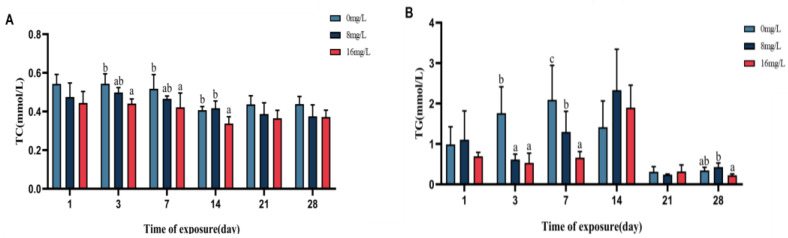
Liver biochemistry of the spotted seabass under 33 °C nitrite stress. (**A**) Total cholesterol (TC) and (**B**) triglyceride (TG). Bars with different letters indicate significant difference (*p* < 0.05). Value is mean ± S.E. (*n* = 3 for treatment).

Changes in hepatic TG and TC contents of the spotted seabass under nitrite stress are shown in Figure 5. Compared to controls, TG showed a marked reduction (*p* < 0.05) in the 16 mg/L group on days 3, 7, and 28 of nitrite stress in the spotted seabass. Meanwhile, no statistically significant intergroup variations (*p* > 0.05) were observed on days 1, 14, and 21 of the stress. With graded increases in nitrite concentration, the TC content of the spotted seabass tended to decrease at d 1, 3, 7, 14, 21, and 28 of nitrite stress, and it was significant (*p* < 0.05) at d 3, 7, and 14 of stress.

### 3.5. Relative Expression of Lipid Metabolism Genes in Liver

**Figure 6:**
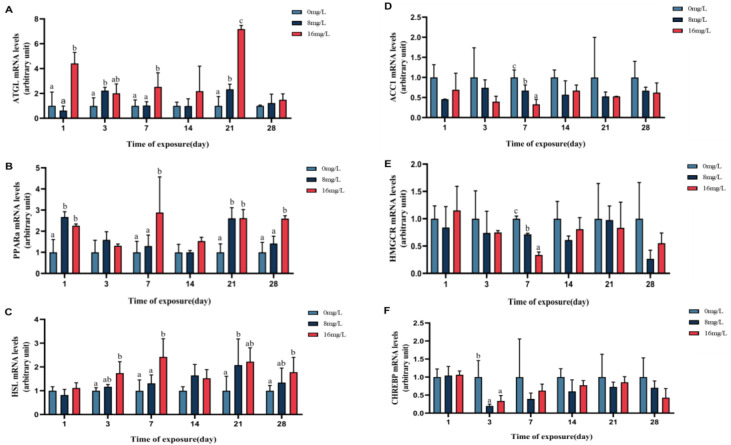
The expressions of liver fat metabolism genes of the spotted seabass under 33 °C nitrite stress. (**A**–**C**) Lipolysis-related genes and (**D**–**F**) liposynthesis-related genes. Bars with different letters indicate significant difference (*p* < 0.05). Value is mean ± S.E. (*n* = 3 for treatment).

The impact of nitrite exposure on the lipolysis (*Atgl*, *Hsl*, and *Ppara*) and lipogenesis (*Acc1*, *Hmgcr*, and *Chrebp*) gene expression of the spotted seabass is shown in Figure 6. When contrasted with unexposed fish, the expression of lipolysis-related genes (*Atgl*, *Hsl*, and *Ppara*) in the high-concentration group (16 mg/L) was significantly upregulated on days 1, 3, 7, and 28 of stress (*p* < 0.05). In contrast, the expression of lipogenesis-related genes (Acc1, Hmgcr, and Chrebp) in both nitrite-exposed groups (8 and 16 mg/L) showed a decreasing trend, with significant downregulation observed for Acc1 and Hmgcr on day 7 and for Chrebp on day 3 (*p* < 0.05).

### 3.6. Glucose Metabolism-Related Indicators in Liver

**Figure 7:**
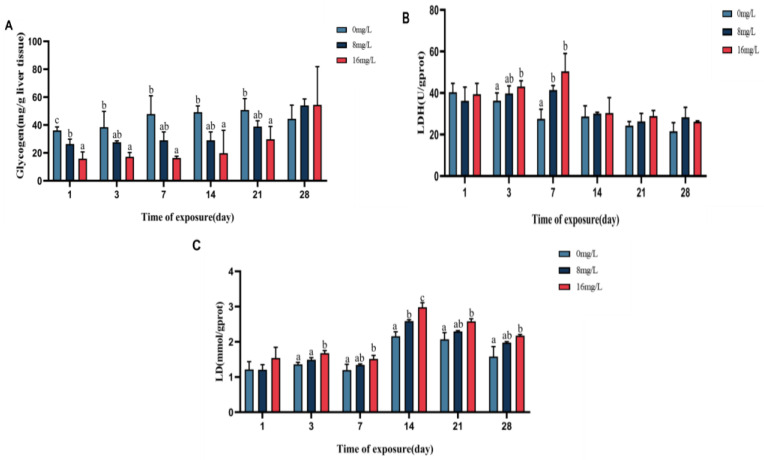
Hepatic glucose metabolism indicators of the spotted seabass under 33 °C nitrite stress. (**A**) Hepatic glycogen (glycogen), (**B**) hepatic lactate dehydrogenase (LDH), and (**C**) hepatic lactic acid (LD). Bars with different letters indicate significant difference (*p* < 0.05). Value is mean ± S.E. (*n* = 3 for treatment).

The impact of nitrite exposure on hepatic GLY, LDH, and LD contents of the spotted seabass is shown in Figure 7. As the concentration of nitrite stress increased, the GLY concentration decreased significantly (*p* < 0.05) on days 1, 3, 7, 14, and 21 of nitrite stress, and statistical analysis revealed no treatment effects on the 28th day (*p* > 0.05). The LDH of the liver of the spotted seabass was significantly (*p* < 0.05) elevated on the 3rd and 7th d of nitrite stress. Meanwhile, no statistically significant intergroup variations were observed (*p* > 0.05) between the experimental groups on days 1, 14, 21, and 28 of the stress. Compared with the control group, the LD concentration in the liver showed an increasing trend with increasing nitrite concentration on days 1, 3, 7, 14, 21, and 28 of stress, and the LD concentration in the liver of the spotted seabass was significantly (*p* < 0.05) higher on days 3, 7, 14, 21, and 28 of nitrite stress.

### 3.7. Metabolomics Analysis

**Figure 8:**
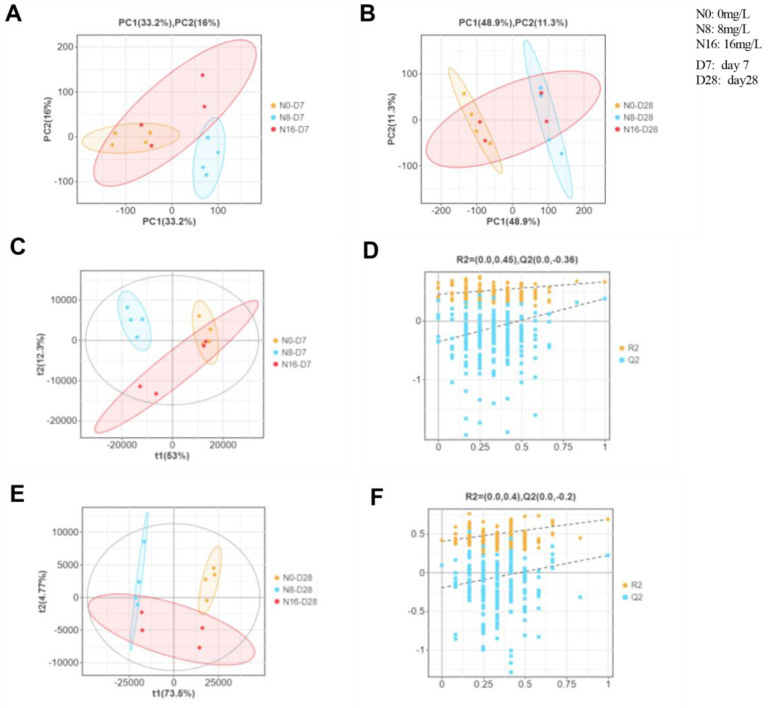
(**A**,**B**) PCA and (**C**–**F**) PLS-DA score plots of liver metabolome of spotted seabass under 33 °C nitrite stress. N0, N8, and N16 represent nitrite concentrations of 0 mg/L, 8 mg/L, and 16 mg/L, respectively. D7 and D28 represent the 7th and 28th days.

**Figure 9:**
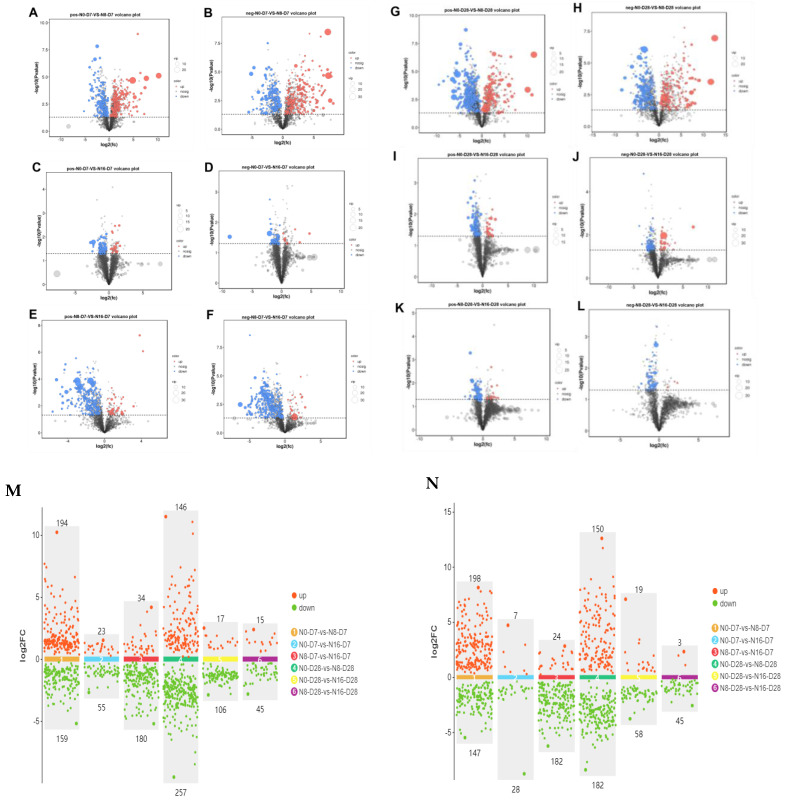
Volcanic diagram of liver metabolites in the spotted seabass under 33 °C nitrite stress. N0, N8, and N16 represent nitrite concentrations of 0 mg/L, 8 mg/L, and 16 mg/L, respectively. D7 and D28 represent the 7th and 28th days. (**A**–**F**) Positive and negative modes of metabolite volcano map on the 7th day, and (**G**–**L**) positive and negative modes of metabolite volcano map on the 28th day. Scatter plots of multiple sets of differences in (**M**) positive and (**N**) negative mode.

PCA and PLS-DA score plots of liver metabolome of the spotted seabass under 33 °C nitrite stress are shown in Figure 8. These PCA and PLS-DA plots show that the 0 mg/L, 8 mg/L, and 16 mg/L concentration groups were significantly separated by regional extent on day 7 and 28, respectively, indicating that nitrite concentration significantly altered the composition of liver differential metabolites in the spotted seabass. The changes that occurred in the abundance liver metabolites in the spotted seabass under 33 °C nitrite stress are shown in Figure 9. The patterns of both up- and downregulated metabolites varied depending on the nitrite concentration and duration of exposure, indicating distinct metabolic responses to stress conditions.

**Figure 10:**
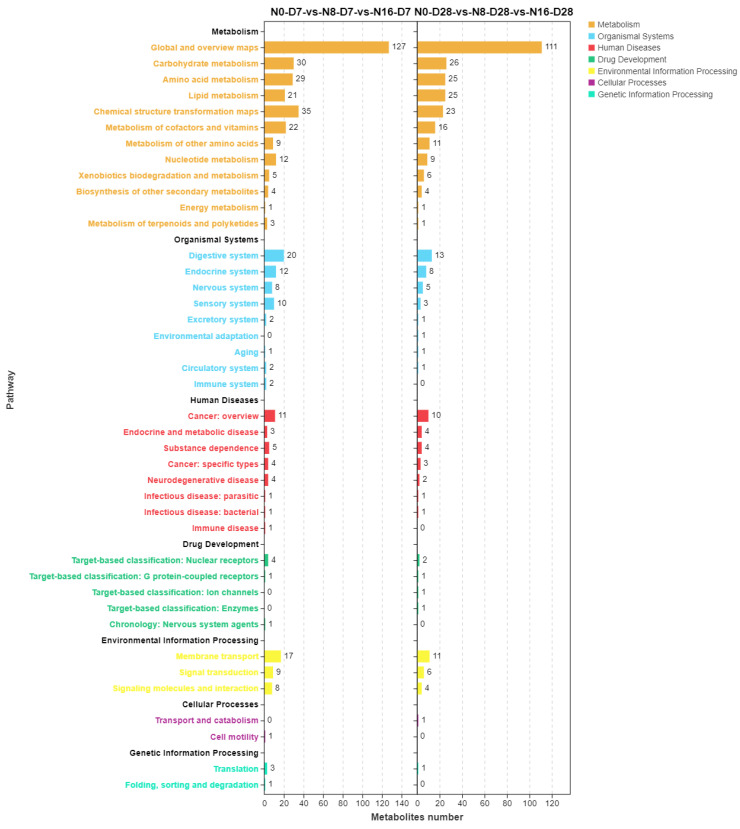
Statistical chart of KEGG enrichment numbers for differences in liver metabolites in the spotted seabass under 33 °C nitrite stress. N0, N8, and N16 represent nitrite concentrations of 0 mg/L, 8 mg/L, and 16 mg/L, respectively. D7 and D28 represent the 7th, and 28th days.

The KEGG classification plot of differential metabolites in the KEGG pathway at different nitrite concentrations (N0, N8, and N16) and time points (D7 and D28) is shown in Figure 10. Nitrite concentration leads to significant enrichment of differential metabolites in the liver in carbohydrate metabolism, amino acid metabolism, lipid metabolism, chemical structure transformation maps, metabolism of cofactors and vitamins, metabolism of other amino acids, and nucleotide metabolism pathways, regardless of whether it is day 7 or day 28.

**Figure 11:**
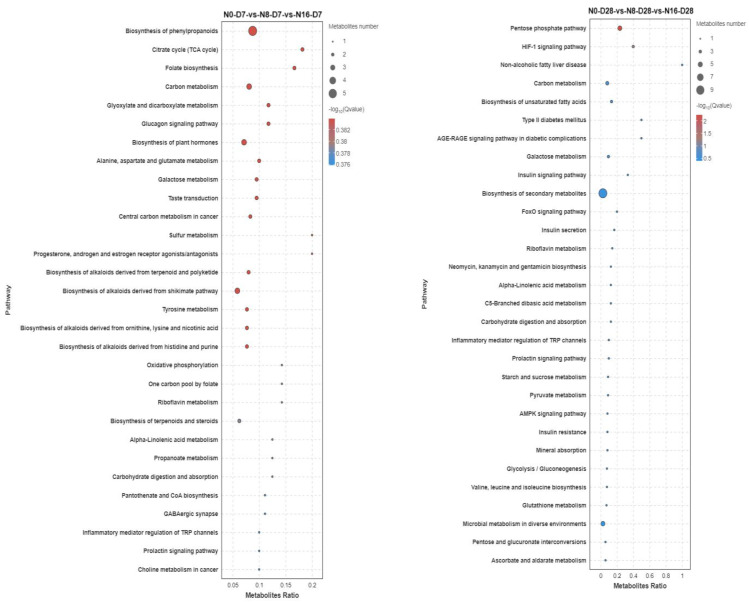
KEGG-enrichment significant bubble plot of liver metabolite differences in the spotted seabass under 33 °C nitrite stress. N0, N8, and N16 represent nitrite concentrations of 0 mg/L, 8 mg/L, and 16 mg/L, respectively. D7 and D28 represent the 7th and 28th days.

The bubble plots show the enrichment of differential metabolites in the KEGG pathway. KEGG enrichment bubble plots on day 7 show that nitrite concentrations resulted in a large enrichment of differential metabolites in biosynthesis of phenylpropanoids; TCA cycle; folate biosynthesis; carbon metabolism; glyoxylate and dicarboxylate metabolism; glucagon signaling pathway; alanine, aspartate, and glutamate metabolism; galactose metabolism; and central carbon metabolism in cancer, among other specific metabolic pathways (Figure 11). The KEGG enrichment bubble plot on day 28 showed that nitrite concentration resulted in a large enrichment of differential metabolites in plasma in specific metabolic pathways, such as the pentose phosphate pathway, the HlF-1 signaling pathway, non-alcoholic fatty liver disease, carbon metabolism, the biosynthesis of unsaturated fatty acids, galactose metabolism, the insulin signaling pathway, the FoxO signaling pathway, insulin secretion, and other specific metabolic pathways.

## 4. Discussion

Elevated nitrite concentrations in aquatic environments have been identified as a major limiting factor for fish growth under intensive aquaculture systems [29,30]. In this study, the actual measured value of the control group is 1 ± 0.5 mg/L; while not absolute zero, it reflects realistic background levels in recirculating aquaculture systems due to inevitable nitrogen cycling. Nitrite exposure substantially impairs growth performance in various fish species [4]. Extensive research has demonstrated that nitrite stress negatively affects the growth, metabolism, and physiological functions of various fish species, including *Labeo rohita*, *Oncorhynchus mykiss*, and *Gadus morhua* [30,31,32]. Similarly, nitrite stress significantly reduced the growth performance of the spotted seabass, with the weight-gain rate in the high-concentration group decreasing by 51% as opposed to unexposed fish. Additionally, when the nitrite concentration in the water body exceeds the self-regulatory threshold of fish, a series of physiological functions in the organism will be disorganized, resulting in fish deaths [4,8]. Comprehensive investigations have revealed that nitrite stress negatively impacts the survival of various fish species, including *O. mykiss*, *Carassius auratus*, and *Gobiocypris rarus* [7]. Similarly, this study found that elevated nitrite concentrations significantly reduced the survival rate of the spotted seabass. This may be attributed to the decrease in hemoglobin levels and the sharp increase in methemoglobin levels due to nitrite stress, leading to hypoxia-induced mortality in fish [16].

Our study demonstrated significant decreases in VSI and HSI of the spotted seabass following nitrite exposure. This phenomenon may be attributed to nitrite-induced hepatic lipid depletion, where the stress response prompts substantial mobilization of stored hepatic lipids for energy production, resulting in hepatic atrophy [33]. Similar results are found in the grass carp (*Ctenopharyngodon idella*) [19] and American bullfrogs (*Aquarana catesbeiana*) [34]. Whole-body compositional analysis revealed a dose-dependent decrease in total lipid content correlating with elevated nitrite concentrations. The crude lipid content in the high-concentration group decreased by 12.03% as opposed to unexposed fish. These findings suggest that nitrite exposure triggers substantial energy expenditure for stress mitigation, thereby impairing nutrient retention mechanisms [35,36]. While lipid will be the main important substance for energy supply, in the environment of nitrite stress, fish may supply energy to the organism by utilizing the decomposition of triglycerides [30,37,38]. We also found that nitrite stress caused different degrees of decreases in triglycerides and total cholesterol at both hepatic and blood plasma in the spotted seabass, which was the main reason for the decreases in crude lipid of the whole body.

During high-level nitrite challenge, the organism of fish usually alters some physiological activities to attenuate nitrite-associated damage [6,39]. A growing body of evidence substantiates that nitrite stress can significantly alter lipid metabolism in fish, though the effects vary by species. In certain species, including juvenile turbot (*Scophthalmus maximus*) and olive flounder (*Paralichthys olivaceus*), nitrite exposure elevates plasma triglyceride and total cholesterol concentrations, indicative of upregulated lipid catabolism to fulfill heightened energy requirements [40]. Conversely, in species like *Pelteobagrus fulvidraco*, *Takifugu rubripes*, and *Larimichthys crocea*, nitrite stress leads to decreased plasma triglyceride and cholesterol levels [41]. Similarly, this study found that blood triglyceride levels in the spotted seabass significantly decreased under nitrite stress. These contrasting results may reflect species-specific metabolic strategies, such as a greater reliance on carbohydrate metabolism for energy, differences in osmoregulatory mechanisms between freshwater and marine species, or localized lipid breakdown in the liver without significant transport to other tissues [6,39]. Overall, nitrite exposure disrupted lipid homeostasis, but the direction and extent of these changes depended on physiological adaptations of species.

The liver is an important energy-supplying organ that reflects fat metabolism through changes in triglyceride and cholesterol levels [19]. In this study, triglyceride content in the high-concentration group significantly decreased during nitrite stress, likely due to enhanced triglyceride breakdown into monoglycerides to meet energy demands, while triglyceride synthesis could not counterbalance [37,42]. High nitrite stress reduced the expression of *Acc1* and *Hmgcr* genes, indicating impaired lipid synthesis and suggesting that prolonged stress may deplete liver fat reserves, making energy supply insufficient to meet physiological demands [20,25]. Key regulators of lipid metabolism, such as *Srebp-1c* (via *Fas* and *Hmgcr* pathways) and Chrebp, were downregulated under nitrite stress, suggesting impaired lipid synthesis [43]. Conversely, the upregulation of Ppara, which promoted fatty acid β-oxidation, and the increased expression of lipolysis-related genes (*Atgl* and *Hsl*) indicated enhanced fat breakdown to supply energy [44]. The results collectively suggested that nitrite stress disrupts lipid homeostasis, driving increased fat utilization for energy while impairing lipid synthesis, ultimately leading to insufficient energy supply under prolonged stress [45].

Nitrite exposure in fish triggers the oxidation of hemoglobin to methemoglobin, resulting in systemic hypoxia, which drives an adaptive metabolic shift toward anaerobic glycolysis [10,46]. Consistent with established hypoxia responses, previous studies document depleted hepatic glycogen stores accompanied by elevated lactate, glycerophosphate, and succinate concentrations [47,48,49]. In this study, medium-to-high nitrite stress significantly decreased hepatic glycogen and increased lactate dehydrogenase activity on days 3 and 7, with hepatic lactate levels rising significantly by day 28. The hepatic glycogen in the high-concentration group decreased by 66.67% as opposed to unexposed fish on days 28. These findings demonstrated that the spotted seabass relied on anaerobic glycolysis to meet energy demands under nitrite-induced hypoxia. Comparable results have been documented in various species, including *Hoplias malabaricus*, *Astronotus ocellatus*, and *Cyprinus carpio*, where nitrite exposure increased liver and plasma lactate levels, reflecting enhanced anaerobic glucose oxidation [50]. Additionally, elevated lactate dehydrogenase (LDH) activity in nitrite-exposed carp suggests increased lactate production as glycolysis shifts to anaerobic pathways [51]. Under nitrite stress, the spotted seabass upregulated lipolysis-related genes and downregulated lipogenesis-related genes to mobilize fat reserves for energy. Simultaneously, the spotted seabass utilized anaerobic glycolysis to compensate for hypoxia, highlighting a dual metabolic adaptation to maintain physiological function.

Liver metabolome analysis provides critical insights into the metabolic adaptations of fish under environmental stress, reflecting complex responses in energy balance and physiological regulation [52,53,54,55,56]. In this study, nitrite stress at 33 °C significantly altered metabolic pathways in the spotted seabass, particularly in carbohydrate, lipid, and amino acid metabolism. The increased energy demand under nitrite stress is likely driven by the need to maintain osmotic balance, as nitrite disrupts ion regulation and osmotic homeostasis in fish. Such physiological compensation necessitates substantial ATP expenditure to sustain ionoregulatory processes in key osmoregulatory tissues, including branchial epithelia, renal tubular systems, and intestinal mucosa. High temperatures exacerbated these challenges by accelerating metabolic rates and increasing the energy required for physiological functions [22,23,24]. Liver metabolomics data also revealed hallmark signatures of HIF-1α activation under nitrite stress. Significant enrichment of glycolytic pathway metabolites and elevated lactate/LDH in Figure 7 and pentose phosphate pathway intermediates, coupled with depletion of TCA cycle metabolites (e.g., succinate and malate). These coordinated shifts reflect HIF-1α-mediated metabolic reprogramming—a conserved adaptive response to hypoxia that prioritizes anaerobic ATP production while suppressing oxidative phosphorylation. Additionally, the enrichment of immune-related pathways suggests tissue damage, further increasing energy demands for repair and immune responses. KEGG enrichment analysis revealed increased enrichment in carbohydrate and lipid metabolism pathways, suggesting that the spotted seabass adapts to nitrite stress by regulating these pathways to meet energy demands. Specifically, the pentose phosphate pathway, unsaturated fatty acid biosynthesis, and glycolysis were significantly enriched, indicating enhanced gluconeogenesis and lipolysis for energy production. This aligns with the observed increase in triglyceride catabolism and upregulation of Pparα, which promotes fatty acid β-oxidation [40,57,58,59]. Additionally, the enrichment of glycometabolic pathways, such as glycolysis, supports the findings of reduced hepatic glycogen and elevated lactate levels, highlighting the importance of glycolysis under nitrite-induced hypoxia. Similar responses have been observed in other species, such as rainbow trout (*Oncorhynchus mykiss*) and zebrafish (*Danio rerio*), where nitrite exposure disrupted hepatic glycolipid metabolism and increased lactate production [51]. The dual stress of nitrite and high temperature likely explains the observed upregulation of lipolysis and glycolysis pathways, enabling the spotted seabass to mobilize energy for osmotic balance, high temperature stress, and tissue repair. These metabolic shifts represent short-term adaptive strategies to mitigate nitrite-induced hypoxia. The upregulation of glycolysis (evidenced by 66.7% hepatic glycogen depletion) and lipid catabolism (12.03% whole-body lipid decrease) provides immediate energy to sustain vital functions under stress. However, these adaptations are unsustainable. Prolonged reliance on anaerobic metabolism depletes glycogen reserves and accumulates toxic lactate, while suppressed lipid synthesis limits long-term energy storage. The pentose phosphate pathway activation indicates compensatory redox balancing, but persistent nitrite exposure overwhelms antioxidant capacity, leading to cellular damage. Despite these efforts, survival rates declined significantly, confirming that adaptations are ultimately overwhelmed by systemic toxicity [18].

Our study reveals that nitrite stress under high temperatures triggers a hypoxia-mediated metabolic shift in the spotted seabass. Nitrite-induced methemoglobinemia reduces oxygen transport, may activate HIF-1α, and diverts energy metabolism toward anaerobic glycolysis, evidenced by hepatic glycogen depletion and lactate accumulation. Concurrently, lipid homeostasis is disrupted through PPARα-mediated upregulation of lipolysis genes (Atgl/Hsl) and suppression of lipogenesis (Acc1/Hmgcr), redirecting energy reserves toward stress adaptation. Metabolomics further confirms TCA cycle suppression and pentose phosphate pathway activation, illustrating how combined stressors reprogram hepatic energy allocation. These interconnected pathways explain the observed growth impairment and metabolic trade-offs.

## 5. Conclusions

In summary, the spotted seabass, when subjected to combined nitrite and thermal stress (33 °C), exhibited reduced growth performance and survival. Nitrite stress triggered increased glycolysis and fatty acid β-oxidation to meet the heightened energy demands. Metabolomics further revealed adaptive strategies, highlighting the regulation of energy metabolism and lipid breakdown to maintain physiological functions under nitrite stress. This study not only elucidates the metabolic disruptions caused by nitrite and high-temperature stress in the spotted seabass but also provides a foundation for developing mitigation strategies in aquaculture, such as optimizing water quality management, formulating stress-resistant feeds enriched with antioxidants or metabolic modulators, and establishing species-specific safety thresholds for nitrite under climate warming scenarios, which could enhance the resilience of aquaculture systems facing increasing environmental stressors. These insights are particularly valuable for regions experiencing rising temperatures, offering practical solutions to mitigate economic losses and improve the sustainability of the fishing industry.

## Figures and Tables

**Table 1:** Primer sequences used for qRT-PCR.

Target Genes	Forward (5′ to 3′)	Reverse (5′ to 3′)	Annealing Temperature (°C)
*Acc1*	AAGGCGGTGGTGATGGATTT	GGCCATGTCGCCTTTGTTTT	60
*Hmgcr*	GACCGTGCATACGGAACAGA	AGTGTGTGGGTTGAGACCG	60
*Chrebp*	GTGACAACGCTCAGCTCTCA	TGATGGCAGAGTTCAGGAGC	60
*Atgl*	CTTCCTCTCCGCAACAAGTC	TGGTGCTGTCTGGAGTGTTC	60
*Hsl*	CGAAACACAGAGACGGTCCA	TCATGACATCTACCAGCCGC	60
*Ppara*	CCGTGCGTGTTTTCACCATT	AGACCAAATACATCGCCCCC	60
*β-actin*	CAACTGGGATGACATGGAGAAG	TTGGCTTTGGGGTTCAGG	60

*Acc1*, acetyl-CoA carboxylase-1; *Hmgcr*, 3-hydroxy-3-methylglutaryl-CoA reductase; *Chrebp*, carbohydrate-responsive element-binding protein; *Atgl*, adipose triglyceride lipase; *Hsl*, hormone-sensitive lipase; *Ppara*, peroxisome proliferator-activated receptor alpha; *β-actin*, beta-actin.

## Data Availability

The data presented in this study are available upon request from the corresponding author.
